# Anti‐Inflammatory and Pro‐Healing Effects of Human Plasma‐Derived Exosomes in a Murine Wound Model

**DOI:** 10.1111/jocd.70559

**Published:** 2025-12-05

**Authors:** Heewoong Yang, Dam Go, Hwansu Kang, Youin Cho, Hyung‐Gi Kim, Jihyun Park, Sang‐Hoon Park, Ki Yong Hong, Hak Chang

**Affiliations:** ^1^ Department of Plastic and Reconstructive Surgery Seoul National University College of Medicine Seoul Republic of Korea; ^2^ Department of Plastic and Reconstructive Surgery Seoul National University Hospital Seoul Republic of Korea; ^3^ Advanced Regenerative Medicine Center ID Hospital Seoul Republic of Korea; ^4^ Department of Chemistry University of Michigan Ann Arbor Michigan USA; ^5^ Department of Plastic Surgery ID Hospital Seoul Republic of Korea

**Keywords:** anti‐inflammatory effects, collagen synthesis, exosomes, plasma‐derived exosomes, wound healing

## Abstract

**Background:**

Exosomes have emerged as key mediators in regenerative medicine because of their ability to facilitate intercellular signaling and promote tissue renewal. Exosomes from various origins have demonstrated efficacy in tissue regeneration.

**Aims:**

To explore the potential of plasma‐derived exosomes (PDEs) in wound healing through in vitro analyses and an in vivo mouse model.

**Patients/Methods:**

PDEs were isolated from blood and characterized using a nanoparticle assay. Human dermal fibroblasts (HDFs) were treated with PDEs at different concentrations to assess cell proliferation, migration, and gene expression. For in vivo evaluation, 8‐mm full‐thickness wounds were created on C57BL/6 mice and treated with either subcutaneous injection or topical smearing of PDEs at low (5 × 10^9^/mL) or high (5 × 10^10^/mL) concentrations. Wound closure was monitored over an 8‐day period, and tissue samples were collected for analysis.

**Results:**

PDEs promoted HDF proliferation and migration in vitro, with significantly higher cell migration rates (38.2, 40.2%) compared to controls (17.8%, *p* < 0.001). Gene expression analysis revealed the upregulation of collagen synthesis markers (*COL1A1*) and the downregulation of degradation markers (*MMP1*). Both subcutaneous injection and topical smearing methods accelerated wound healing in vivo, with closure rates of 91.8%–96.5% in treated groups versus 70.9%–72.6% in controls by day 8. Treatment increased the expression of regenerative markers *(Fgf1*, *Fn1*) while reducing the levels of inflammatory markers (*Il6, Ptgs2*).

**Conclusion:**

PDEs promote wound healing by enhancing cell proliferation, stimulating collagen synthesis, and modulating inflammatory responses. Both subcutaneous injection and topical smearing were effective.

## 1 Introduction

In recent years, exosomes have emerged as significant components in regenerative medicine due to their role in intercellular communication and tissue repair. Initially considered cellular waste products, exosomes gained recognition in the 2000s when studies demonstrated their function in transferring bioactive molecules—proteins, lipids, and RNAs—between cells [1]. During the 2010s, advances in isolation and characterization techniques further revealed their therapeutic potential, particularly as mediators of paracrine signaling in stem cell therapies [2].

Mesenchymal stem cell (MSC)‐derived exosomes promote angiogenesis, modulate inflammation, and facilitate tissue regeneration, offering a promising cell‐free alternative to conventional stem cell transplantation [3]. Their ability to deliver therapeutic cargo, such as RNAs and growth factors, with lower immunogenicity compared to whole‐cell therapies has positioned exosomes as valuable tools in tissue engineering and regenerative applications, including wound healing, cardiac repair, and bone regeneration [4].

Exosomes isolated from human umbilical cord blood endothelial progenitor cells (EPCs) enhance endothelial cell proliferation, migration, and angiogenesis, thereby accelerating wound closure in diabetic rat models [5]. Similarly, exosomes derived from blood outgrowth endothelial cells (BOECs) promote vascular network formation and neovascularization, leading to improved wound healing outcomes in murine models [6]. Human umbilical cord blood‐derived exosomes have also been shown to modulate inflammatory pathways and enhance tissue repair mechanisms, resulting in increased cell migration and collagen deposition [7]. In diabetic wound models, human umbilical vein endothelial cell‐derived exosomes have been shown to improve fibroblast function and angiogenesis, leading to enhanced wound healing [8].

However, practical challenges associated with the procurement and cultivation of EPCs from umbilical cord blood EPCs or BOECs, such as limited cell yield, donor‐to‐donor variability, specialized laboratory requirements, and prolonged cell expansion times, further complicate their widespread clinical applicability [9, 10, 11]. Compared with cell‐derived exosomes, plasma‐derived exosomes (PDEs) offer several advantages, including easier accessibility, abundant availability, and cost‐effectiveness, as they can be obtained directly from blood without the need for complex cell isolation or cultivation procedures. Additionally, pooling samples from multiple donors may reduce donor variability and improve standardization, thereby facilitating clinical translation [12, 13].

In this study, we aimed to characterize the functions of PDEs through both in vitro and in vivo analyses. Specifically, we investigated the cellular uptake of PDEs using fluorescence microscopy and evaluated their effects on collagen synthesis and anti‐inflammatory gene expression.

## 2 Materials and Methods

### 2.1 Institutional Review Board (IRB) Approval

Human blood samples were obtained in accordance with the principles of the Declaration of Helsinki and with the approval of our institute (Institutional Review Board No. 1506‐136‐683). All procedures involving human‐derived materials were conducted in accordance with relevant ethical guidelines and institutional regulations.

### 2.2 Exosome Isolation and Nanoparticle Tracking Analysis

For exosome isolation, 10 mL of blood was collected from a single volunteer and centrifuged at 3500 rpm for 10 min to obtain plasma. The plasma was then subjected to a second centrifugation at 10 000× *g* for 10 min to collect the supernatant, which was named platelet‐poor plasma (PPP). Exosomes were isolated from 1 mL of PPP at room temperature (20°C) using a 35 nm qEV1 size‐exclusion column (IZON Science Ltd., Addington, New Zealand) according to the manufacturer's protocol. The initial 4.7 mL of buffer separated from the column was discarded, and the subsequent 2.8 mL of buffer containing exosomes was collected for analysis. The collected exosomes were designated as PDEs. To clarify the necessary yield, this isolation process was repeated multiple times on the plasma from the single volunteer until the requisite quantity of exosomes was acquired for the following experiments. Specifically, four isolations were performed.

The isolated exosomes were diluted 1:10 000 in normal saline, and their size and distribution were analyzed using NanoSight Pro (Malvern Panalytical, Malvern, UK). The analysis was performed under the following conditions: a syringe pump flow rate of 3 μL/min, a camera gain of 4.8, and 750 capture frames. Five captures were obtained to calculate the average, and the data were analyzed using NS XPLORER software (version 1.1.0.6, Malvern Panalytical).

### 2.3 Culture of Human Dermal Fibroblasts (HDFs)

Human dermal fibroblasts (HDFs; Promocell, Heidelberg, Germany) were cultured in Dulbecco's Modified Eagle Medium (DMEM; Gibco, Waltham, USA) supplemented with 10% fetal bovine serum (FBS; Gibco) and 1% penicillin–streptomycin (P/S; Gibco) in 100‐mm culture dishes (SPL, Pyeongtaek, Korea). Cells were maintained in a humidified incubator at 37°C with 5% CO_2_. The culture medium was replaced every 2–3 days, and experiments were conducted when the cells reached 80%–90% confluence.

### 2.4 Cell Proliferation Assay

Cell proliferation was assessed using CCK‐8 reagent (Dojindo, Kumamoto, Japan). HDFs were seeded at 5 × 10^3^ cells per well in 96‐well plates (SPL) and cultured for 12 h. The PDEs were serially diluted 1:1 over five steps, reducing the concentration from 100% to 3.13%. Each diluted concentration was applied to the HDFs. After 48 h of incubation, the proliferation assay was performed following the manufacturer's protocol. The absorbance was measured at 450 nm using a Spectramax ID3 microplate reader (Molecular Devices, San Jose, USA), and the data were analyzed using SoftMax Pro 7.1 software (Molecular Devices).

For the proliferation assay, HDFs were treated with two concentrations of exosomes. The high‐concentration group (designated as Exo‐H) was defined as 5 × 10^10^ particles/mL—the highest concentration at which exosomes retained their therapeutic efficacy—whereas the low‐concentration group (designated as Exo‐L) was defined as 5 × 10^9^ particles/mL (one‐tenth of the highest concentration).

### 2.5 Exosome and Cell Staining Results

Exosome staining was performed using PKH26 (CAS:154214‐55‐8; Sigma‐Aldrich, St. Louis, USA) for exosomes, carboxy‐fluorescein succinimidyl ester (CFSE; Invitrogen, Waltham, USA) for the cytoplasm, and 4′,6‐diamidino‐2‐phenylindole (DAPI; Invitrogen) for nuclei, following the manufacturer's instructions. HDFs were seeded at 2.0 × 10^5^ cells/well in 6‐well plates (SPL) and incubated for 24 h. Subsequently, PKH26‐labeled exosomes were added to the cells at a concentration of 2.5 × 10^10^ particles for 24 h. Cells were fixed with 4% paraformaldehyde (Biosesang, Yongin, Korea) for 30 min and washed twice with phosphate‐buffered saline (PBS; Welgene, Daegu, Korea). The cytoplasm was stained with CFSE, followed by two additional washes with PBS. The nuclei were stained with DAPI, and the cells were washed twice with PBS. After the addition of 2 mL of PBS, images were captured using a fluorescence microscope (CKX53; Olympus, Tokyo, Japan) before the cells dried.

### 2.6 Scratch Assay for Cell Migration Analysis

HDFs were seeded at 5.0 × 10^5^ cells per well in 6‐well plates and cultured for 12 h. Subsequently, the cells were treated with 10 μg/mL mitomycin C (Abcam, Cambridge, UK) in serum‐free DMEM for 3 h. Next, a scratch was created in each well using SPLScar (SPL), and PDEs were added. The cells were incubated for 24 h. Images were captured at 0 and 24 h using a fluorescence microscope, and the extent of cell migration (scratch closure) was analyzed using ImageJ software (NIH, Bethesda, MD, USA).

### 2.7 RNA Extraction and Real‐Time Polymerase Chain Reaction (PCR) Analysis

HDFs were seeded at a density of 2.5 × 10^5^ cells per well in 6‐well plates and incubated for 12 h. The cells were subsequently treated with PDEs for 48 h, before harvesting. To analyze anti‐inflammatory gene expression, inflammation was induced using lipopolysaccharide (LPS).

RNA was extracted from the collected samples using RNAiso Plus (Takara, Shiga, Japan) according to the manufacturer's protocol. The RNA concentration was measured using a Spectramax ID3 microplate reader (Molecular Devices). Reverse transcription PCR was performed using PrimeScript RT Master Mix (Takara), and the synthesized cDNA was used for PCR analysis. Real‐time PCR was performed using the TB Green Premix Ex Taq II (Takara) reagent on a QuantStudio 3 (Applied Biosystems, Waltham, USA) system for amplification and analysis. The target genes and markers, along with their corresponding primers are listed in Supporting Content No. 1.

### 2.8 Animal Experiments

Forty‐two 8‐week‐old male C57BL/6 mice (Koatech, Pyeongtaek, Korea) were procured and used in the study. The appropriate number of experimental mice per group (*n* = 7) was determined using statistical power analysis. The animals were acclimatized to the laboratory environment for 7 days before the start of the experiment. Housing conditions were maintained at a temperature of 21°C–25°C and a humidity level between 40% and 60%. Mice were provided ad libitum access to solid food and water. All animal procedures were approved by the Institutional Animal Care and Use Committee (No. 24‐0124‐S1A1) of Seoul National University Hospital.

### 2.9 Wound Model and Treatment

Mice were anesthetized using isoflurane (KyongBo Pharmaceutical, Asan, Korea) at a concentration of 2.5%–3%. The dorsal region hair was shaved using an electric clipper, and complete depilation was achieved with a depilatory cream (Ildong, Seoul, Korea). An 8‐mm wound was created on the dorsal side of each mouse using a punch biopsy tool (Kai Medical, Seki, Japan). A wound splint (Gracebio, Bend, USA) was applied and secured with sutures to prevent wound contraction.

Exosome treatments were administered at two concentrations—Exo‐L (low dose) and Exo‐H (high dose)—as defined in the in vitro cell proliferation study. Six groups of seven mice were established according to the route of administration and dosage: “SC‐Low” and “SC‐High” labeled mice were treated with subcutaneous injections of Exo‐L and Exo‐H, while “SM‐Low” and “SM‐High” labeled mice received topical smearing of the corresponding doses. Two additional groups, “SC control” and “SM control”, received saline via subcutaneous injection or topical smearing.

For the exosome‐treated groups, 100 μL of exosome solutions with their respective concentrations were administered three times on days 0, 2, and 4. In the subcutaneous injection groups, the solution was divided into six separate injections around the wound site and delivered using an insulin syringe (BD, Franklin Lakes, USA). In the topical smearing groups, the entire 100 μL was applied directly to the wound to ensure complete absorption without spillage. The control groups received the same volume of saline using the same application method.

**FIGURE 1:**
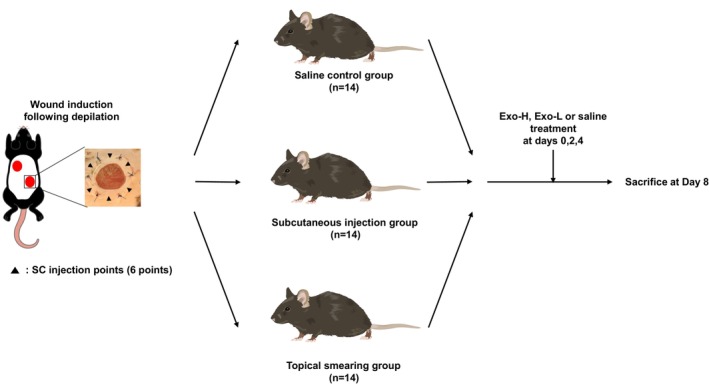
Schematic of the animal model study. Overview of the in vivo experimental design of wound healing, including treatment groups, administration routes, and time points for analysis. Exo‐H, 5 × 10^10^ exosome concentration; Exo‐L, 5 × 10^9^ exosome concentration; SC, subcutaneous.

Wound healing was monitored and photographed at 2‐day intervals until day 8 post‐induction. The degree of wound closure was analyzed using ImageJ (Figure 1).

### 2.10 Histological Analysis

On the eighth day after wounding, skin tissues from the control, subcutaneous injection, and topical smearing groups were harvested. The skin tissue samples were fixed in 10% formalin (Biosolution, Seoul, Korea) for 48 h, followed by dehydration and embedding in paraffin blocks. Tissue sections of 4 μm thickness were prepared and stained using the hematoxylin and eosin (H&E) staining method. The results were analyzed using a CKX53 microscope (OLYMPUS). A Bead Ruptor Elite (Revvity, Waltham, USA) was used to lyse re‐epithelialized mouse skin tissue. Subsequently, RNA extraction and real‐time PCR were performed following the same protocol as that used for HDF cells.

### 2.11 Statistical Analysis

The results are expressed as the mean ± standard deviation (mean ± SD) of three independent replicate experiments. Statistical analysis was performed using the Statistical Package for the Social Sciences (version 20.0.0, IBM, New York, USA), and analysis of variance was performed to retrieve *p*‐values. Statistical significance was determined as *p < 0.05, **p < 0.01, and ***p < 0.001.

## 3 Results

### 3.1 Exosome Isolation and Characterization

**FIGURE 2:**
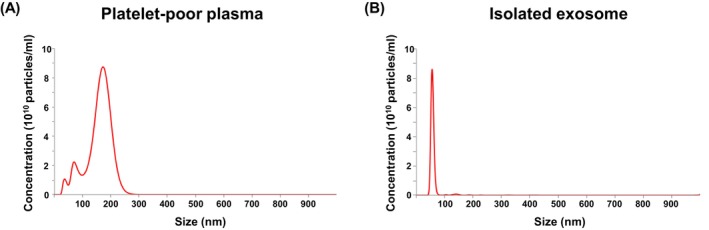
Characterization of plasma‐derived exosomes (PDEs). (A) Nanoparticle tracking analysis (NTA) of platelet‐poor plasma (PPP) reveals multiple particle populations, with a dominant peak at 180 nm. (B) After size exclusion chromatography, purified PDEs show a single major peak at 180 nm, indicating successful isolation with over 98% purity.

PPP isolated from whole blood exhibited multiple peaks in size distribution, with the most prominent peak at 180 nm, followed by secondary peaks at 70 and 35 nm. Following size‐exclusion chromatography, nanoparticle tracking analysis using NanoSight Pro confirmed the successful isolation of PDEs, with a purity exceeding 98% (Figure 2).

### 3.2 Effects on HDF Proliferation

**FIGURE 3:**
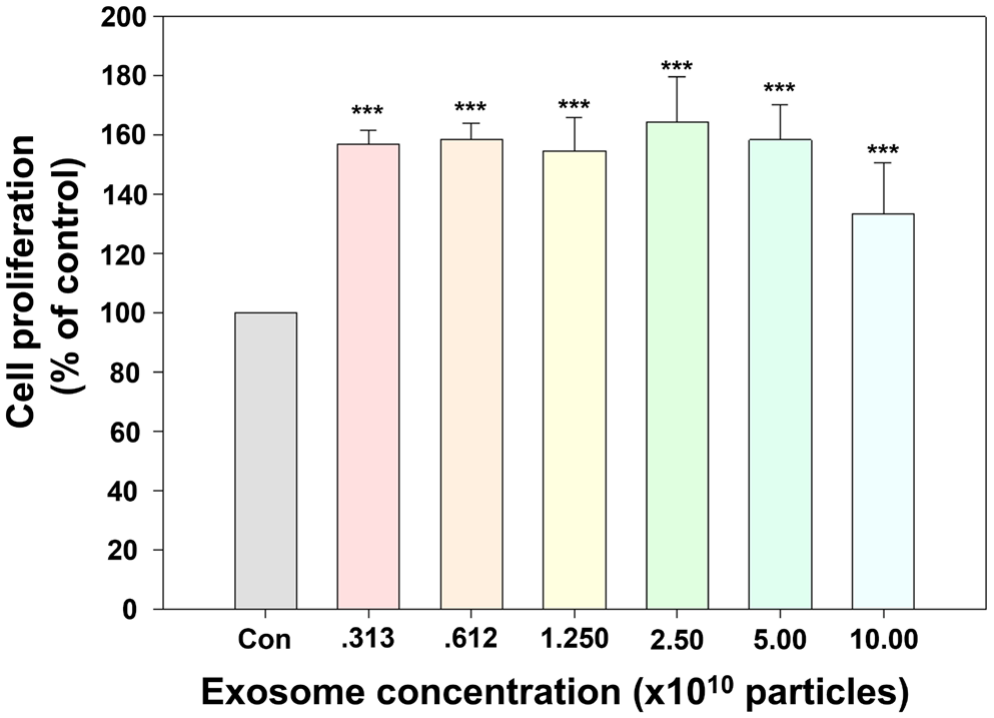
Effect of exosome treatment on proliferation of human dermal fibroblasts (HDFs). Cell viability assessed using a CCK‐8 assay revealed significantly increased proliferation in all exosome‐treated groups compared with the control (***p < 0.001). The highest proliferation rate was observed at 2.5 × 10^10^ particles. Data are presented as percentages relative to the control. Con, saline‐treated control group.

Compared to the control group, exosome treatment significantly enhanced HDF proliferation at all tested concentrations (*p* < 0.001). The highest proliferation rate was observed at 2.5 × 10^10^ particles, reaching 164.2% relative to the control group. As the increase plateaued beyond 5 × 10^10^ particles, concentrations of 5 × 10^9^ and 5 × 10^10^ were selected for animal experiments as Exo‐L and Exo‐H, respectively (Figure 3).

### 3.3 Cellular Uptake and Migration Assay

**FIGURE 4:**
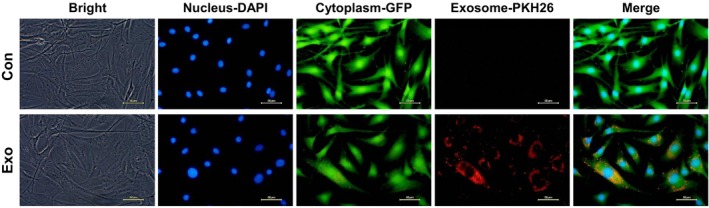
Intracellular uptake of exosomes by human dermal fibroblasts (HDFs). Confocal microscopy images showing PKH26‐labeled exosomes (red) successfully internalized into the cytoplasm (green) of GFP‐expressing HDFs, confirming cellular uptake.

**FIGURE 5:**
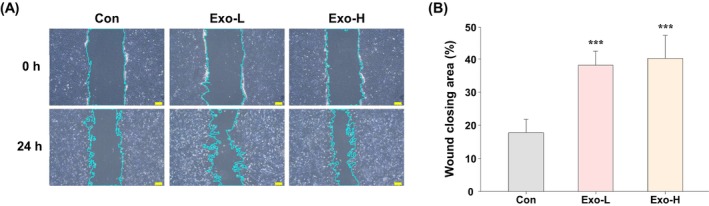
Wound healing enhancement in an in vitro scratch assay. (A) Representative microscopic images of scratch assays in the control, Exo‐L, and Exo‐H groups (scale bar = 100 μm). (B) Quantitative analysis of wound closure shows significantly enhanced migration in the exosome‐treated groups compared with the control group (***p < 0.001). Con, control; Exo‐H, 5 × 10^10^ exosome concentration; Exo‐L: 5 × 10^9^ exosome concentration.

PKH26‐labeled exosomes were efficiently internalized into HDFs, as visualized in GFP‐expressing cells (Figure 4). In the scratch assay, wound closure rates were 17.8%, 38.2%, and 40.2% in the control, Exo‐L, and Exo‐H groups, respectively (*p* < 0.001 vs. control for both), indicating significantly enhanced migratory activity following exosome uptake (Figure 5).

### 3.4 Analysis of Collagen‐Related Gene and Marker Expression in HDFs

**FIGURE 6:**
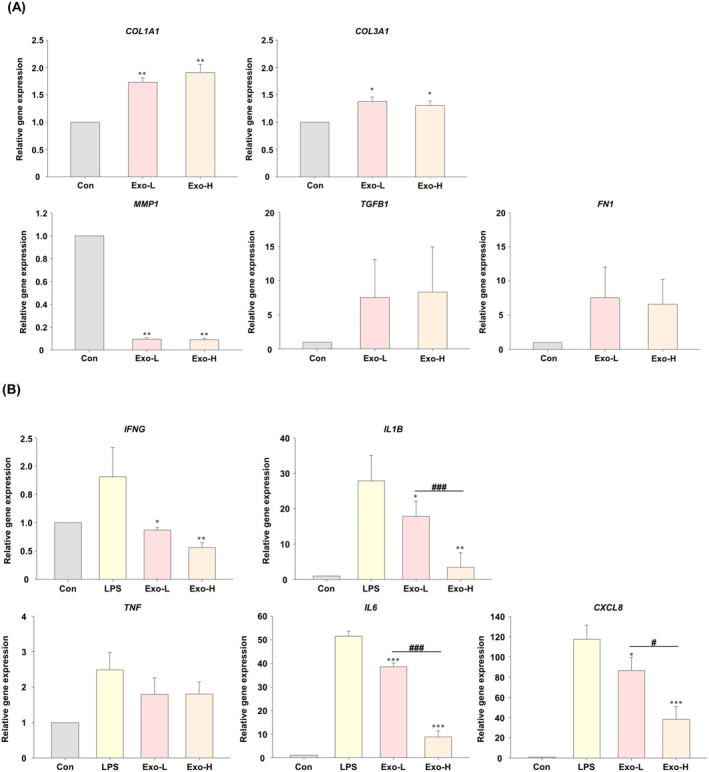
Gene and marker expression in human dermal fibroblasts (HDFs) following exosome treatment. Real‐time polymerase chain reaction results evaluating cellular responses to exosome treatment (*p < 0.05, **p < 0.01, ***p < 0.001 versus control; #*p* < 0.05, ##*p* < 0.01, ###*p* < 0.001 Exo‐L vs. Exo‐H). (A) COL1A1, COL3A1, and MMP1 expression levels indicate enhanced collagen synthesis and reduced matrix degradation. Increases in TGFB1 and FN1 also suggest improved wound healing, but the difference was not statistically significant. (B) Expression of inflammatory marker genes (*IFNG, IL1B, TNF, IL6*, and *CXCL8*) in lipopolysaccharide (LPS)‐induced HDFs demonstrate that exosome treatment significantly downregulated proinflammatory cytokines, but not *TNF*, in a dose‐dependent manner. Con, control; Exo‐H, 5 × 10^10^ exosome concentration; Exo‐L, 5 × 10^9^ exosome concentration.

Exosome treatment led to the upregulation of collagen synthesis‐related markers and suppression of degradation‐related genes. *COL1A1* expression increased 1.73‐fold in the Exo‐L group and 1.91‐fold in the Exo‐H group (*p* < 0.001 vs. control). *COL3A1* expression was elevated to 1.38‐ and 1.30‐fold in the Exo‐L and Exo‐H groups, respectively (*p* < 0.05 vs. control). *MMP1* expression was reduced to 0.09‐fold in both groups (*p* < 0.001) (Figure 6A). *TGFB1* expression was substantially amplified, though statistical significance was not achieved due to high variability. *FN1* expression increased in both groups, 7.57‐fold in the Exo‐L group and 6.60‐fold in the Exo‐H group, but with borderline statistical significance in only the Exo‐H group (*p* = 0.052).

### 3.5 Modulation of the Inflammatory Response in HDFs

In an LPS‐induced inflammatory model, exosome treatment attenuated the expression of pro‐inflammatory genes. *IFNG* levels were reduced from 1.81‐fold in the control group to 0.87‐fold in the Exo‐L group (*p* < 0.05) and 0.56‐fold in the Exo‐H group (*p* < 0.01). *IL6* expression decreased from 51.53‐fold to 38.56‐fold (Exo‐L, *p* < 0.001) and 8.77‐fold (Exo‐H, *p* < 0.001), with significant differences between the Exo‐L and Exo‐H groups (*p* < 0.001). The *TNF* levels were slightly reduced (Exo‐L: *p* = 0.056; Exo‐H: *p* = 0.059), indicating borderline significance. *CXCL8* expression was lowered from 117.64‐fold in the control group to 86.54‐fold in the Exo‐L group (*p* < 0.05) and 38.22‐fold in the Exo‐H group (*p* < 0.001), with significant differences between the two exosome‐treated groups (*p* < 0.05). The *IL1B* levels decreased to 17.83‐fold in the Exo‐L group (*p* < 0.05) and 3.49‐fold in the Exo‐H group (*p* < 0.01), with a significant intergroup difference (*p* < 0.05) (Figure 6B).

### 3.6 In Vivo Wound Healing Effects

**FIGURE 7:**
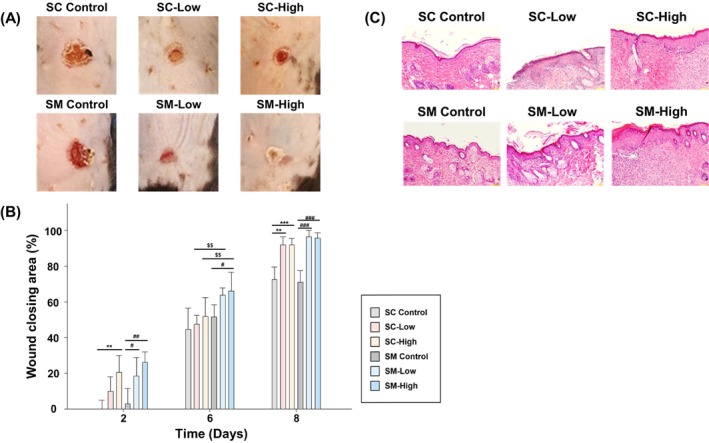
In vivo wound healing and histological analysis. (A) Representative images of dorsal wounds from each treatment group captured on days 2, 6, and 8. (B) Quantitative wound closure analysis over time demonstrates significantly accelerated healing in exosome‐treated groups (***p < 0.001, **p < 0.01, *p < 0.05 vs. SC control; ###*p* < 0.001, ##*p* < 0.01, #*p* < 0.05 vs. SM control; $$*p* < 0.01 SC vs. SM). (C) H&E‐stained skin tissue sections show increased epidermal and dermal thickness after exosome treatment. Scale bars and group‐wise comparisons are presented. H&E, hematoxylin and eosin; SC, subcutaneous injection; SC‐Control, saline treated group via subcutaneous injection; SC‐High, 5 × 10^10^ exosome concentration treated group via subcutaneous injection; SC‐Low, 5 × 10^9^ exosome concentration treated group via subcutaneous injection; SM, topical smearing; SM‐Control, saline treated group via topical smearing; SM‐High, 5 × 10^10^ exosome concentration treated group via topical smearing; SM‐Low, 5 × 10^9^ exosome concentration treated group via topical smearing.

Wound closure was evaluated on days 2, 6, and 8 in both SC and SM models (Figure 7A). On days 2, 6, and 8, the SC control group exhibited wound closure areas of −0.16%, 44.5%, and 72.6%; the SC‐Low group exhibited wound closure areas of 9.88%, 47.5%, and 91.8% (*p* < 0.01 on day 8); and the SC‐High group exhibited wound closure areas of 20.4% (*p* < 0.001), 51.8%, and 91.9% (*p* < 0.001), respectively. On the same days, the wound closure areas of the SM control group were 2.82%, 51.6%, and 70.9%; those of the SM‐Low group were 18.5% (*p* < 0.05), 63.6%, and 96.5% (*p* < 0.001); and those of the SM‐High group were 26.1% (*p* < 0.01), 66.0%, and 95.6% (*p* < 0.001), respectively (Figure 7B).

On day 6, the route of administration had a significant impact on wound healing, with both low‐ and high‐dose SM groups exhibiting superior healing compared with their SC counterparts (*p* < 0.01), supporting the enhanced efficacy of topical delivery at intermediate stages. By day 8, all exosome‐treated groups significantly outperformed the controls, with comparable effectiveness between the SC and SM routes.

Histological analysis confirmed increased epidermal and dermal thicknesses in the exosome‐treated groups (SC control: 51.81 μm; SC‐Low: 139.40 μm; SC‐High: 180.87 μm; SM control: 70.74 μm; SM‐Low: 132.59 μm; SM‐High: 173.13 μm; *p* < 0.01) (Figure 7C).

### 3.7 Analysis of Collagen‐Related Gene and Marker Expression in Animal Models

**FIGURE 8:**
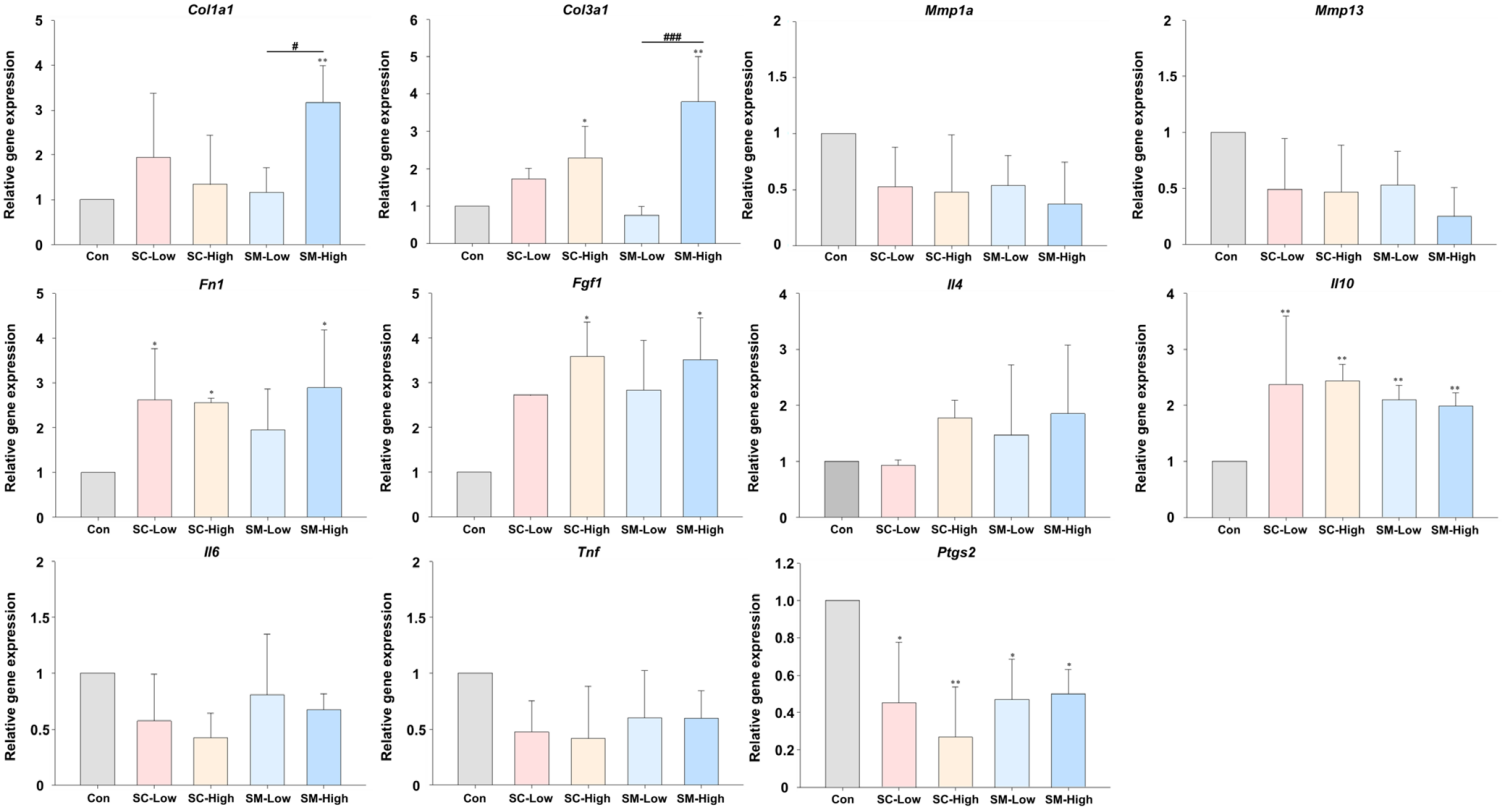
Gene and marker expression in the wound tissues of the animal model. Real‐time polymerase chain reaction analysis of tissue collected on day 8 post‐treatment (*p < 0.05, **p < 0.01, ***p < 0.001; #*p* < 0.05, ##*p* < 0.01, ###*p* < 0.001 Exo‐L vs. Exo‐H). Upregulated expression levels of *Col1a1* and *Col3a1* and downregulation of *Mmp1a* and *Mmp13* indicate increased collagen formation and decreased breakdown rate, although not significantly. The upregulation of the wound healing‐related factors *Fgf1* and *Fn1* suggests improved wound healing. Changes in the expression of the inflammatory cytokine genes *Il10* and *Ptgs2* demonstrate the anti‐inflammatory effects of exosome treatment. The increase in *Il4* and decrease in *Il6* and *Tnf* also suggest anti‐inflammatory effects, albeit not reaching statistical significance. SC‐Control, saline treated group via subcutaneous injection; SC‐High, 5 × 10^10^ exosome concentration treated group via subcutaneous injection; SC‐Low, 5 × 10^9^ exosome concentration treated group via subcutaneous injection; SM‐Control, saline treated group via topical smearing; SM‐High, 5 × 10^10^ exosome concentration treated group via topical smearing; SM‐Low, 5 × 10^9^ exosome concentration treated group via topical smearing.

*Col1a1* expression was elevated by 1.95‐, 1.34‐, 1.16‐, and 3.17‐fold in the SC‐Low, SC‐High, SM‐Low, and SM‐High groups, respectively (*p* < 0.01 vs. control; *p* < 0.05 between SM‐Low and SM‐High mice). *Col3a1* was similarly upregulated (SC‐Low: 1.72‐fold; SC‐High: 2.28‐fold, *p* < 0.05; SM‐High: 3.79‐fold, *p* < 0.01), while SM‐Low exhibited a modest downregulation (0.75‐fold, *p* < 0.001 vs. SM‐High). *Mmp1a* and *Mmp13* were downregulated across all treatment groups, although the differences were not statistically significant. (Figure 8).

### 3.8 Modulation of Wound‐Healing Factors

*Fn1* expression increased in all groups: SC‐Low (2.62‐fold, *p* < 0.05), SC‐High (2.56‐fold, *p* < 0.05), SM‐Low (1.96‐fold), and SM‐High (2.89‐fold, *p* < 0.05). *Fgf1* expression followed a similar trend, with significant elevation observed in SC‐High (3.59‐fold, *p* < 0.05) and SM‐High (3.51‐fold, *p* < 0.05) groups, whereas expression in SC‐Low and SM‐Low increased by 2.72‐ and 2.83‐fold, respectively (Figure 8).

### 3.9 Modulation of the Inflammatory Response in Model Mice

Anti‐inflammatory cytokines, including *Il4*, increased across all groups, although the differences were not statistically significant. *Il10* was significantly upregulated in all treatment conditions (SC‐Low: 2.37‐fold; SC‐High: 2.44‐fold; SM‐Low: 2.10‐fold; SM‐High: 1.99‐fold; all *p* < 0.01 vs. control). Pro‐inflammatory mediators were suppressed, as evidenced by decreased levels of *Il6* and *Tnf*, although the changes did not reach statistical significance. *Ptgs2* levels were significantly reduced in all groups (SC‐Low: 0.45‐fold, *p* < 0.05; SC‐High: 0.27‐fold, *p* < 0.01; SM‐Low: 0.47‐fold, *p* < 0.05; SM‐High: 0.50‐fold, *p* < 0.05) (Figure 8).

## 4 Discussion

In this study, we aimed to investigate the effects of exosome treatment on wound healing and associated molecular changes using both in vitro and in vivo approaches. In the animal experiment, through an extended gene expression analysis involving more than ten key genes, we observed elevated levels of *Fn1* and *Col1a1*, along with the suppression of pro‐inflammatory mediators such as *Tnf* and *Ptgs2*. These findings suggest that PDEs contribute to both inflammation suppression and extracellular matrix (ECM) remodeling, aligning with processes activated during the proliferative and remodeling phases of wound healing. These results are consistent with those of previous studies, further supporting our findings [14, 15, 16, 17].

Furthermore, our findings underscore the importance of ECM regulation in the healing process. Type I collagen is the dominant form of collagen, providing tensile strength and structure to mature skin. In contrast, Type III collagen is more abundant during the early stages of wound healing, forming a scaffold that is later gradually replaced by Type I collagen [18]. The observed modulation of both COL1A1 and COL3A1 by PDE treatment highlights a concerted effort to regulate the quality and speed of ECM deposition, optimizing the transition from early granulation tissue to mature dermal tissue.

Previous studies have examined the therapeutic potential of exosomes in wound repair. However, as noted in a systematic review by Prasai et al., heterogeneity in exosome origin and isolation protocols remains a challenge in drawing consistent conclusions. Nonetheless, the review supports the idea that exosomes promote healing across all stages of the repair process [14].

A distinguishing feature of the current study is the use of PDEs, which are less commonly investigated compared with MSC‐derived exosomes. However, there are a few precedents—such as the study by Chen et al., which reported that serum‐derived exosomes (SDEs), a broader term adapting PDEs, enhanced wound healing in diabetic mice [19]. Our study expands upon this by incorporating a broader gene expression panel using RT‐PCR and concurrently evaluating the wound closure rate, vascularity, and gene expression under controlled conditions. Furthermore, we employed both topical smearing and subcutaneous injection routes of exosome delivery at two concentrations, allowing for a direct comparison of administration methods within a unified experimental setup.

Specifically, there have been reports that PDEs are capable of immunomodulatory functions. Li et al. demonstrated that PDEs exerted this function by promoting M1 polarization in macrophages, creating a more inflammatory environment [20]. PDEs have also been shown to enhance angiogenesis by upregulating key macrophage factors, such as VEGFA and NDRG1. This process is linked to the previously noted potential of PDEs for ECM remodeling, showing that PDEs enhance angiogenesis and tissue regeneration in damaged areas.

Interestingly, topical smearing yielded superior outcomes in certain cases compared with subcutaneous injection. Previous studies have shown that the topical application of exosomes is effective in wound healing and possibly skin rejuvenation [21, 22, 23]. Although this observation requires further validation, the noninvasive nature of topical delivery offers practical advantages, including ease of administration, reduced patient discomfort, and greater accessibility in clinical settings. With the increasing interest in exosome‐based topical products, our findings support the continued exploration of this route, particularly for application in wound care and aesthetics.

From a translational standpoint, PDEs may provide a more accessible alternative to cell‐based therapies. Although MSC‐derived exosomes have been widely studied, their clinical use is often complicated by production costs, batch variability, and ethical or regulatory considerations [24]. In contrast, PDEs are easier to source, have fewer ethical concerns, and are more amenable to standardization. These advantages position PDEs as a promising candidate for broader clinical application, especially in contexts requiring scalable and cell‐free therapeutic approaches [25, 26].

Despite our study's strengths, certain limitations should be acknowledged. First, although our results suggest that PDEs have beneficial effects on wound healing, we did not conduct an in‐depth mechanistic study of the underlying molecular mechanisms. Nevertheless, previous studies have indicated that SDEs may modulate key signaling pathways, such as Akt/mTOR and TGF‐β, and promote anti‐inflammatory macrophage polarization [27, 28]. These mechanisms may help explain the observed improvements in tissue repair. Second, we used healthy C57BL/6 mice rather than pathological models, such as diabetic or impaired healing models, which may limit our findings' direct applicability to clinical cases involving delayed wound healing. Future studies incorporating disease‐relevant models are necessary to extend the clinical relevance of our findings. The last recognized limitation of this study is the reliance on plasma collected from a single volunteer. Although multiple isolation steps were performed to ensure sufficient exosome yield, and given that the concentration used was determined to be the minimum effective concentration for the assay, this single source may not fully capture the inherent biological variation in exosome concentration that exists within the general population. Future studies should be expanded to include a larger cohort of healthy participants.

Given the accessibility and safety profile of PDEs, further studies should explore their potential for clinical translation, including dose optimization, delivery methods, and long‐term efficacy.

## 5 Conclusions

Using an animal model, our study demonstrates that exosomes significantly contribute to wound healing. Both subcutaneous injection and topical smearing were effective in enhancing wound recovery. The demonstrated efficacy of topical smearing for delivering PDEs presents a highly practical, non‐invasive, and effective strategy for clinical application in wound care, strongly affirming its superiority as a localized delivery route. Detailed comparisons of various inflammatory and regenerative markers confirmed the anti‐inflammatory and wound‐healing effects of exosome treatment. These findings highlight the promising therapeutic potential of exosomes in accelerating tissue repair and mitigating inflammation.

## Author Contributions

Heewoong Yang: experiment, investigation, methodology, writing – original content, editing and review. Dam Go: experiment, data curation, formal analysis, investigation, methodology, writing – methods. Youin Cho, Hyung‐Gi Kim, Jihyun Park, Hwansu Kang: experiment, investigation, methodology, validation, data curation. Sang‐Hoon Park, Ki Yong Hong, Hak Chang: conceptualisation, formal analysis, funding acquisition, investigation, methodology, project administration, resource, supervision, validation, writing – review and editing.

## Ethics Statement

This study was conducted in accordance with the principles of the Declaration of Helsinki and was approved by the Institutional Review Board of Seoul National University Hospital (IRB No. 1506‐136‐683).

## Conflicts of Interest

The authors declare no conflicts of interest.

## Supporting information


**Data S1:** jocd70559‐sup‐0001‐Supinfo.pdf.

## Data Availability

Research data are not shared.
