# Supplementary material for: Anti‐Inflammatory and Pro‐Healing Effects of Human Plasma‐Derived Exosomes in a Murine Wound Model
Source: J Cosmet Dermatol. 2025 Dec 5;24(12):e70559. doi: 10.1111/jocd.70559 (PMC12679673; doi:10.1111/jocd.70559)
Supplement: Supplementary file 1 — Data S1: jocd70559‐sup‐0001‐Supinfo.pdf. [file JOCD-24-e70559-s001.pdf]

Supplementary file No. 1 : Table of used human genes and primers

	Gene name	NCBI Reference Sequence	Forward Primer	Reverse Primer
Human	*GAPDH*	NM_001256799	CAACAGCCTCAAGATCATCAG	GAGTCCTTCCACGATACCAA
	COL1A1	NM_000088	CCTGTCTGCTTCCTGTAA	GGTTGCTTGTCTGTTTCC
	COL3A1	NM_000090	GCGGATAGAGATGTCTGGAA	CGAGTAGGAGCAGTTGGA
	MMP1	NM_001145938	TGATGAAGCAGCCCAGAT	TCAATCCTGTAGGTCAGATGT
	TGFB1	NM_000660	GCGTCTGCTGAGGCTCAAGTTA	TTGCTGAGGTATCGCCAGGAAT
	FN1	NM_001306129	CAGCCTCTGGTTCAGACTGC	GCTTGTGGGTGTGACCTGAG
	IFNG	NM_000619	TGAATGTCCAACGCAAAGCAATAC	GACCTCGAAACAGCATCTGACTC
	IL6	NM_000600	AGTCCTGATCCAGTTCCT	GCAGAATGAGATGAGTTGTC
	TNF	NM_000594	AATGGCGTGGAGCTGAGAG	TTGAAGAGGACCTGGGAGTAGAT
	CXCL8	NM_000584	TCTCTTGGCAGCCTTCCTGATT	GGGTGGAAAGGTTTGGAGTATGTC
	IL1B	NM_000576	CCTGTACGATCACTGAACTG	CACCACTTGTTGCTCCAT

	Gene name	NCBI Reference Sequence	Forward Primer	Reverse Primer
Mouse	*Gapdh*	NM_001289726	ACCTGCCAAGTATGATGA	GGAGTTGCTGTTGAAGTC
	Col1a1	NM_007742	GTGAGACAGGCGAACAAG	CCAGGAGAACCAGGAGAA
	Col3a1	NM_009930	CACAAGGATTACAAGGCATACC	CAGGAGCACCGACTTCAC
	Mmp1a	NM_032006	GGGCTGAAAGTGACTGGAAA	AGGATTGTTGTGAGTAATGGCATA
	Mmp13	NM_008607	ACAGTTGACAGGCTCCGAGAA	CCACATCAGGCACTCCACATC
	Fgf1	NM_010197	GTCAAACTACAACTCCAAGCAGAA	CCAGCAGCCGTCCATCTT
	Fn1	NM_001276408	CCTGCCGAATGTAGATGA	GCTTCCTGTCCTGTAGAG
	Il4	NM_021283	GAATGTACCAGGAGCCATATC	TGTTCTTCGTTGCTGTGAG
	Il10	NM_010548	GGTTGCCAAGCCTTATCG	GCATCCTGAGGGTCTTCA
	Il6	NM_001314054	TCCATCCAGTTGCCTTCT	GGAGTGGTATCCTCTGTGAA
	Tnf	NM_001278601	GTGGAACTGGCAGAAGAG	TGAGAAGAGGCTGAGACATA
	Ptgs2	NM_011198	GCAACGACATGGAGACAATC	CTGTGAGGACAACGAGGAA
